# Immersive Tai Chi for Home-Based Exercise in Older Adults: Usability and Feasibility Study

**DOI:** 10.2196/79453

**Published:** 2026-01-28

**Authors:** XiaCheng Song, Nazlena Mohamad Ali, Mohamad Hidir Mhd Salim, Muhammad Yudhi Rezaldi

**Affiliations:** 1 Institute of Visual Informatics, National University of Malaysia, Aras 1, Bangi, Selangor, 43600, Malaysia, 60 3 8927 2413; 2 Research Centre for Data and Information Sciences, National Research and Innovation Agency, Bandung, Indonesia; 3 Faculty of Creative Industry, Telkom University, Bandung, Indonesia

**Keywords:** virtual reality, video games, aged, Tai Chi, usability testing, motion sickness

## Abstract

**Background:**

Longer life expectancy makes physical exercise crucial for active aging. However, adherence to traditional exercise among community-dwelling older adults is generally low. Virtual reality (VR) and mixed reality (MR) Tai Chi exergames, as novel health promotion tools, show significant potential, particularly for older adults exercising in a home setting.

**Objective:**

This study aimed to evaluate the usability and feasibility of a VR and MR Tai Chi exergame for community-dwelling older adults, focusing on subjective experience, physiological comfort, and objective interaction performance. The study also explored the relationships between key usability factors and sought to quantify links between objective accuracy and subjective experience (the Game Experience Questionnaire or Virtual Reality Sickness Questionnaire [VRSQ]) to inform choices of display mode, feedback strength, and session length.

**Methods:**

Of the 86 community-dwelling older adults recruited for this study, data from 70 participants were considered valid after an initial screening, during which 16 (18.6%) were excluded due to issues with VR adaptation. Participants were sequentially assigned in a rotating order to 1 of 4 variants (VR/MR×soothing/intense) to balance exposure; however, primary analyses were preplanned to be collapsed across variants, focusing on whole-sample usability and feasibility rather than confirmatory between-group hypotheses. The primary outcome measures included the Game Experience Questionnaire, VRSQ, and objective gameplay logs.

**Results:**

The VR/MR Tai Chi game demonstrated good overall usability and acceptability among the screened participants. Subjective experience was highly positive, with median scores for “positive affect” (median 4.0) and “competence” (median 3.8) being significantly high, whereas the median for “Challenge” (median 1.4) was significantly low (*P*<.001 for all). Physiological comfort in the postscreening sample was acceptable, with the most common mild symptoms being dizziness with eyes closed (20.0%) and vertigo (18.6%), both of low severity; however, the initial exclusion of 18.6% of participants due to VR discomfort is noteworthy. Therefore, generalizability is limited because the analyzed sample overrepresents older adults who tolerate immersive displays. Accuracy showed significant positive correlations with flow (ρ=0.342) and competence (ρ=0.322), whereas the VRSQ total score was significantly negatively correlated with positive affect (ρ=−0.334, *P*=.005).

**Conclusions:**

Tai Chi exergames based on immersive technologies offer a feasible and attractive pathway for promoting physical exercise among community-dwelling older adults, particularly within the home environment, supporting the goal of aging in place. As a single, laboratory-based session, the reported satisfaction may partly reflect a novelty effect; therefore, longer-term, home-based follow-ups are needed to assess durability. Analysis of the key usability factors provides guidance for specific design choices, while also indicating directions for future research, such as longitudinal evaluations, extension to more diverse populations, and application in real-world home settings.

## Introduction

The trend of people getting older around the world is accelerating. By 2050, there will be more than 2 billion people over the age of 60 years, and older adults will make up a larger share of the world’s population, especially in developing areas [1]. This change in the population puts more pressure on the economy and shows how important programs such as “Healthy Aging” and letting older people “age in place” are. Regular exercise is very important for healthy aging [2]. It helps keep people healthy, helps them stay independent, makes them feel better mentally, and improves their quality of life [3]. These benefits make exercise a good and useful way to help older people all over the world. However, older adults often face numerous barriers that make it hard for them to participate in traditional exercise programs. These problems include not being motivated, being afraid of falling, and not being able to get to the right facilities [4]. These challenges show that conventional exercise models often fail to meet the emotional and experiential needs of older adults. Recent studies suggest that digitally delivered, personalized interventions, such as programs incorporating wearable technology, remote coaching, and motivational messaging, can significantly improve adherence and reduce fear of falling [5,6]. This points to the need for approaches that improve enjoyment and intrinsic motivation in older adults.

To tackle this issue, immersive exergames that combine virtual reality (VR) and mixed reality (MR) technologies are a viable solution. This research concentrates on the integration of Tai Chi with VR/MR technologies. Tai Chi is particularly suitable for older adults due to its proven efficacy in enhancing dynamic balance and substantially decreasing both the frequency of falls and the fear of falling, which is a significant psychological impediment to physical activity engagement in this demographic [7]. These advantages render Tai Chi a pragmatic and psychologically beneficial approach for enhancing mobility and self-assurance in older adults. VR/MR Tai Chi exergames are meant to be a safe, easy, and fun way to work out at home. These exergames can address many limitations of traditional exercise by providing virtual practice environments that are controllable. They may also boost intrinsic motivation by enhancing feelings of autonomy and competence. Although extended reality technologies hold substantial promise for helping older adults, several challenges remain, such as the high cost of equipment, steep learning curves, and cybersickness. Notably, previous research has emphasized that cybersickness is a frequent and underestimated barrier that may lead to user dropout and restrict broader adoption in unsupervised home settings [8,9]. These usability and safety issues should be addressed before extended reality solutions can be widely implemented among older populations.

Although VR and MR exergames show promise for helping older adults avoid falling, there is insufficient systematic research comparing how different display modes and feedback systems affect this group. Some early evidence suggests that augmented reality, a type of MR, may be better for older adults because it gives them a better sense of their body and space, which makes them feel less likely to fall [10]. This points to a research gap in comprehending the preferences and reactions of older users to various immersive environments. Recognizing the distinct interactive experiences of older adults is essential for effective design, particularly in unsupervised home contexts. Hence, distinguishing between VR and MR modes goes beyond being a mere technical matter; it has direct implications for the safety, comfort, and acceptance of these technologies among older adults using them independently at home.

This work provides evidence to guide the development of immersive health care applications to enable older individuals to practice independently at home, a key component within the “aging in place” plan. With professional guidance lacking within home contexts, intrinsic motivational abilities and efficacy and safety of the game within itself become paramount [11]. Thus, this work’s investigation into aspects such as cybersickness and “accuracy-competence paradox” remains a key focal point within this work to aid in determining if such technology can be disseminated and leveraged effectively.

Specifically, this study will seek to answer the following core questions:

1. Overall usability and acceptance: How does age affect subjective experience (eg, “empowerment” feelings), physiological comfort (ie, cybersickness symptom occurrence), and objective interaction efficacy (eg, the “accuracy-competence paradox”) while playing the VR/MR Tai Chi game?
2. Internal links between fundamental factors: How does objective gameplay performance (eg, pointing precision) influence subjective psychological experiences (eg, flow, competence)? Do users’ physiological symptoms of discomfort affect positive emotions? Are positive emotions correlated to users’ inherent traits, such as age?

Through systematic investigation of these questions, this study aimed to contribute empirical evidence to be used in designing immersive health applications among older people [12].

## Methods

### Ethical Considerations

The study received ethics approval and adhered to guidelines for research involving human participants.

#### Research Ethics Approval

This study received ethics approval from the Institute of Visual Informatics, Universiti Kebangsaan Malaysia. The approval was granted on July 2, 2024, with the reference number UKM.IVI.600-4/6/P130610.

#### Informed Consent

All participants provided written informed consent before the start of any experimental procedures. The researchers clarified the study’s objectives, methods, possible risks (including cybersickness symptoms), and potential benefits. They also made it clear to participants that they could withdraw from the study at any point without facing any consequences.

#### Privacy and Confidentiality

All personally identifiable information and harvested data were fully anonymized to ensure respondent privacy. Data were stored securely on a password-protected server, and only central research team staff had access.

#### Compensation

Participants did not receive any monetary or nonmonetary compensation for their participation. Their involvement in this study was entirely voluntary.

### Study Design

#### Overview

This study was designed as a pilot study using a 2×2 factorial design (display mode: VR/MR×feedback: soothing/intense) to effectively explore the feasibility of varying exergame configurations before a large trial. Participants were sequentially assigned to 1 of the 4 conditions using a rotating 1:1:1:1 schedule upon enrollment. The primary goal of this multicondition design was not to conduct formal hypothesis testing between groups, which would require a much larger sample. Instead, its purpose was to ensure that our usability and feasibility assessment covered a range of potential interaction styles. This approach allows us to identify any major negative outcomes (eg, excessive cybersickness or frustration) that might be unique to a specific condition, providing critical insights for optimizing a single, refined protocol for future, larger-scale home-based studies.

#### Recruitment, Screening, and Final Sample

A research team initially recruited 86 community-dwelling older people who were capable of living independently, via media such as community promotions at community centers and newspaper advertising locally. Such a process was used to maintain the sample’s characteristics closely aligned with those of the target end user group for future home-based VR/MR Tai Chi exergames [13].

In this pilot phase of research, screening led to a dropout of 16 participants (16/86, 18.6% of the original sample) who could not accommodate to the virtual environment or who experienced severe dizziness. Notably, this screening process suggests that the final sample was skewed to some degree to VR tolerance, that is, comprised individuals better predisposed to accommodate virtual environment exposures. This characteristic is relevant in estimating the extent to which the findings generalize to a broader community of older individuals.

We performed an analysis of the 16 participants who were excluded due to a failure to adapt to VR. This group comprised 11 (68.8%) women and 5 (31.2%) men, a gender distribution similar to that of the retained sample. However, a systematic difference was evident in age: 43.8% (7/16) of participants excluded were aged older than 75 years, a proportion more than double that of the sample maintained (15/70, 21.4%). This finding suggests that older age could be a determining factor in intolerance to VR in this population.

#### Inclusion and Exclusion Criteria

The aforementioned recruitment and screening processes adhered to clearly defined criteria. The inclusion criterion was enrollment of participants aged 60 years and older [14,15]. Exclusion criteria encompassed any known adverse reactions to VR experiences and severe cardiorespiratory or musculoskeletal diseases that would significantly impede participation in the experimental procedures, severe vision loss, and other health conditions unsuitable for the use of immersive devices (eg, vertigo and epilepsy).

Self-report alone was considered insufficient; therefore, a brief experiential screening was recommended before unsupervised use.

#### Demographic Characteristics, Health Status, and Exercise Habits

Data regarding demographic information, health background, and daily exercise habits were collected from the 70 valid participants using a self-developed “basic information questionnaire.” The detailed characteristics are presented in Table 1.

The 70 valid participants included in the final analysis exhibited certain demographic tendencies: they were predominantly female (52/70, 74.29%) and largely comprised “younger” older adults aged 60 to 75 years (55/70, collectively 78.56%). In terms of health status, although a considerable proportion (40/70, 57.14%) self-reported chronic conditions such as cardiopulmonary diseases, they were also individuals who actively managed their health, with a high percentage (66/70, 94.29%) undergoing regular physical examinations. This characteristic of “living actively with illness” suggests that the sample group might hold a more open attitude toward health-promoting interventions, but it also necessitates that such interventions ensure safety and appropriateness.

**Table 1.** Participant demographic characteristics, health status, and exercise habits (N=70).

Characteristics	Values, n (%)
Sex	
Male	18 (26)
Female	52 (74)
Age distribution (y)	
60‐65	32 (46)
65‐70	11 (16)
71‐75	12 (17)
75‐80	11 (16)
>80	4 (6)
Height distribution (cm)	
140‐150	13 (19)
150‐160	36 (51)
160‐170	20 (29)
>170	1 (1)
Weight distribution (kg)	
30‐40	2 (3)
40‐50	8 (11)
50‐60	36 (51)
60‐70	18 (26)
70‐80	4 (6)
>80	2 (3)
Self-reported illness status	
No reported cardiopulmonary illness	30 (43)
With cardiopulmonary disease	40 (57)
Regular physical examination	
Yes	66 (94)
No	4 (6)
Weekly exercise frequency	
Exercise daily	66 (94)
Exercise 3‐5 times per week	1 (1)
Rarely exercise	3 (4)

This pilot group reported very high levels of physical activity, such that the vast majority (66/70, 94.29%) used a daily exercise regimen and participated in a broad range of activities, such as walking, square dancing, and Tai Chi. Overall, this sample is a subgroup of predominantly female, aged between 60 and 75 years, who exercise on a regular basis, approach health care management in an active manner, and have good tolerance to VR environment (as a consequence of preliminary screening that eliminated 18.6% (16/86) who reported dizziness while experiencing VR). These characteristics are relevant to explain why this sample reports such a high level of interest and positive response to the VR Tai Chi game. While these same characteristics will limit how widely the investigation can be generalized to broader older groups, such as those who exercise less or who possess poorer VR tolerance.

### VR Tai Chi Exergame and Experimental Equipment

#### Intervention and Equipment

The VR Tai Chi exergame used in this study was designed for older individuals. Its fundamentals can be equally analyzed using the MDA (mechanics, dynamics, and aesthetics) approach [16,17].

#### Mechanics

It guides users through 16 selected Tai Chi exercises. It is interactive via a head-mounted display (HMD) and hand controllers reacting to head and hand movements. Users must synchronize their pose to 3 hotspots (head and both hands) provided in the on-screen instruction. A movement only proceeds after a successful success window, and a movement can save up to 3 hotspot holds (left hand, right hand, and HMD) before advancing. If users complete the entire sequence of 16 movements ahead of schedule, they will repeat the sequence immediately, beginning with the first movement. This generous success condition was a conscious design choice intended to provide a sense of accomplishment. It has 2 display modes, VR and MR, and can come equipped with either soothing or upbeat music and vibration feedback. Additionally, players can skip the current move or pause at will during gameplay.

#### Dynamics

Playing involves players’ efforts at mirroring exemplified postures, receiving immediate multisensory feedback, and regulating progression during a trial lasting a maximum of 5 minutes. One key dynamic reveals itself in the gamification mechanics: participants can choose to meet only the bare minimum threshold needed for progress (“single hotspot for 3 s”) to progress effortlessly or shoot for immaculate matching across all hotspots to progress to a higher “accuracy” level. This allows participants to balance challenge against victory on a preference and ability level. It is this dynamic that later results regarding user experience build upon.

#### Aesthetics

The game aims to evoke multidimensional aesthetic experiences, such as a “sense of competence” from successfully completing movements, a “sense of challenge” from improving performance, feelings of “relaxation or motivation” engendered by different musical feedback, and a “sense of immersion” provided by the VR/MR environments. These subjective experiences are intended to encourage physical exercise in older users in an engaging manner, and their achievement was assessed using the Game Experience Questionnaire (GEQ) [18,19]. Design philosophy appears to be interested in desirable affective effects and perceived competence corresponding to self-determination theory principles, such that satisfying the need for competence can improve intrinsic motivation.

#### Experimental Equipment

This study used the Meta Quest 3 (Meta Platforms, Inc) HMD for presenting both VR and MR conditions [20,21]. The choice of specific, commercially available hardware, such as the Meta Quest 3, enhances the transparency and potential replicability of the study.

#### Activity Area

Exercise took place in an area of approximately 5 m², comparable to a typical living room, to ensure home feasibility and safety [22,23].

### Experimental Procedure

Group allocation followed a prespecified rotating schedule (1:1:1:1) prepared before data collection; participants had no influence on assignment.

#### Experimental Preparation and Adaptation Training

When participants arrived at the laboratory, they first completed an informed consent process and a general information questionnaire. Next, a short adaptation training session was completed to enable participants (and especially those without any training in VR/MR) to become familiar with both the immersive environment and how to use the equipment. This adaptation step is a valuable step in acclimating older adults to new technology, such as VR, because it can be used to help reduce technology anxiety and achieve a minimum baseline level of comfort and competence in how to use the equipment before beginning the intervention [24,25].

#### Exergame Intervention

**Figure 1.**
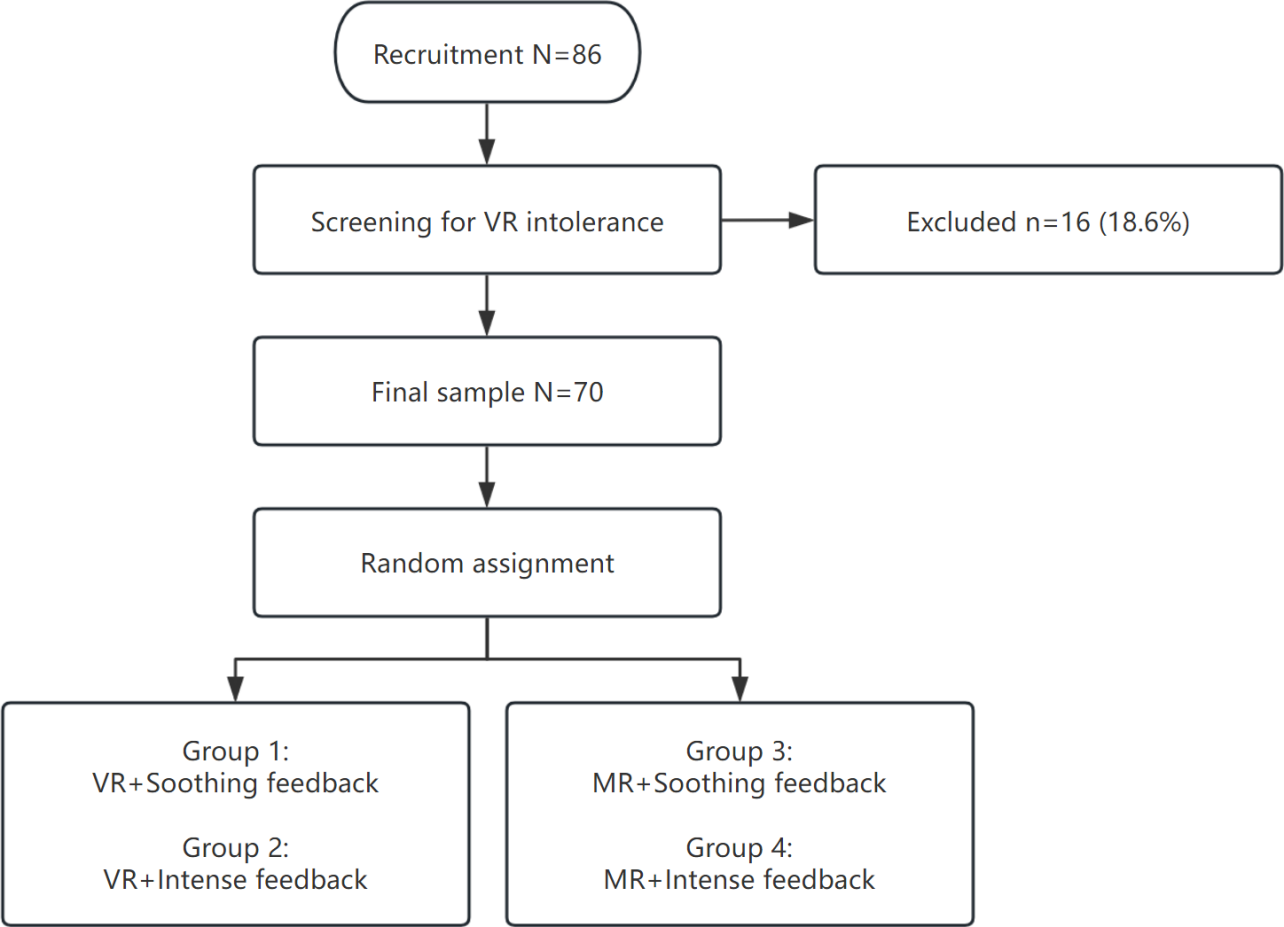
Participant Flow and 2×2 Study Design. MR: mixed reality; VR: virtual reality.

A visual summary of the participant flow and 2×2 design (VR/MR×soothing/intense) appears in Figure 1. This approach ensured that group sizes remained balanced and that potential confounding factors, such as personal preference or prior experience, were minimized. Participants followed a fixed 16-movement sequence; each 5-minute session could end before all movements were finished due to the time limit or restart at the first movement if the sequence was completed early. After this orientation, each participant performed 2 formal 5-minute gameplay sessions under their assigned experimental condition (VR/MR×soothing/intense feedback). The better performance out of the 2 sessions was recorded for analysis. All single-session metrics (eg, posture, accuracy) were computed per session; the higher of the 2 sessions was retained for analysis. This procedure ensured that every participant experienced the complete set of movements while also minimizing the impact of first-trial unfamiliarity on the final outcome [26].

**Figure 2.**
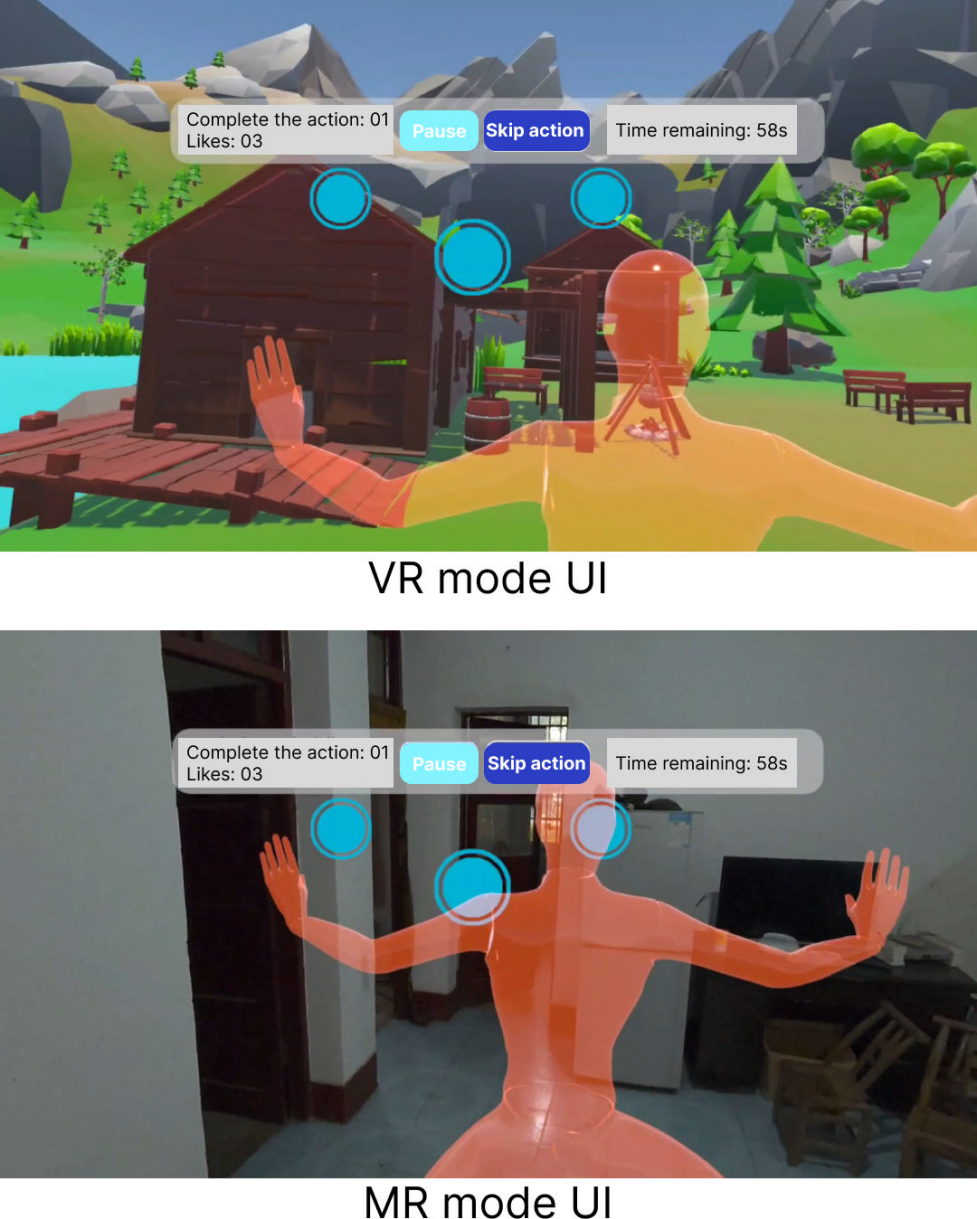
Screenshots of the VR and MR display modes. MR: mixed reality; UI: user interface; VR: virtual reality.

VR provided a fully virtual scene; MR overlaid guidance onto the real room (Figure 2). “Soothing” used gentle cues; “intense” used more salient multimodal cues; task rules were identical [27].

#### Safety and Supervision

The experimental protocol was observed and supervised in a one-on-one capacity by trained researchers to be able to control and respond to any new sensory stimulus or sensation of disorientation. Subjects were informed they could withdraw from participation at any time if they experienced any type of discomfort or abnormal level of fatigability. Such precautions are particularly relevant in research among older individuals and potentially disorienting technologies such as VR. To regulate fatigue and potential dizziness between sessions, exposure was scheduled in 2 approximately 5-minute sessions, and participants could rest and pause whenever needed.

This was a single-visit, supervised laboratory study, consisting of the 2 gameplay blocks described earlier, with no home-based or longitudinal follow-up. Postsession questionnaires captured immediate responses; repeated exposures to control for novelty were not implemented.

### Outcome Measures

#### Overview

This research took a multidimensional perspective to assess usability and feasibility, where primary data were drawn from:

1. GEQ [18]: An adapted 32-item version of the basic module of the GEQ (skipping item 3 in the original 33-item) was used to measure seven aspects of game experience: flow, competence, positive affect, negative affect, tension/annoyance, challenge, and sensory/imaginative immersion. Item 3 (“I was interested in the game’s story”) was excluded from the component immersion because our own game did not have a story. Component flow and competence scores were of particular importance to intrinsic motivation. Component scores were calculated as the means of their items on a scale ranging from 0 to 4.
2. The Virtual Reality Sickness Questionnaire (VRSQ) [28]: It was used to detect and quantify physiological symptom indicators of distress that users may experience while controlling VR/MR hardware, such as eye strain, nausea, headache, disorientation, and dizziness. It is a 9-item measure scored on a 0- to 3-point scale, and the VRSQ total score was calculated as the mean of these items, which provided a final score between 0 and 3. It has been shown to be a valid measure in the original investigation of the VRSQ for cybersickness because it was very highly correlated with existing motion sickness indices.
3. Objective game performance logs: Primary objective metrics were derived from automatically collected gameplay data provided by the game’s backend system. They were “game score,” “movement accuracy,” “mean time per round,” “task completion rate,” “movement level (extracted from hand movement and head movement trajectory)," and “cumulative game time.” These data were direct evidence to substantiate the assessment of whether users could use the game in an efficient manner.

We took an integrated approach, triangulating subjective experience (GEQ), physical comfort (VRSQ), and actual performance (game logs) to provide a sound foundation for a unified and rigorous assessment of the game’s usability and feasibility.

We adopted a target engineering level of 80% movement precision to be used as an internal and exploration benchmarking.

#### Psychometric Properties of Outcome Scales

Internal consistency was evaluated on the present sample using Cronbach α and Spearman-Brown split-half coefficients. As several components (tension/annoyance, challenge, and negative affect) exhibited pronounced floor effects with near-zero variance, internal consistency coefficients for these scales were not estimable; for these, we report distributional summaries only. Reliability results for all measures are presented in Table 2. Per the GEQ guidelines, we dropped the story-related immersion item (item 3), leaving 5 items for this component; reliability values in Table 2 reflect this adapted set.

**Table 2.** Internal consistency of Game Experience Questionnaire and Virtual Reality Sickness Questionnaire (VRSQ) subscales.

Measure	k (items)	Cronbach α	Split-half (Spearman-Brown)
Sensory and imaginative immersion	5^a^	0.657	0.583
Flow	5	0.546	0.539
Tension/annoyance	3	N/A^b^ (floor effect)	N/A^b^ (floor effect)
Challenge	5	N/A^b^ (floor effect)	N/A^b^ (floor effect)
Negative affect	4	N/A^b^ (floor effect)	N/A^b^ (floor effect)
Positive affect	5	0.86	0.85
VRSQ total	9	0.782	0.689

a Game Experience Questionnaire core scored 0‐4; immersion used items 12, 18, 19, 27, 30 (item 3 removed). Split-half denotes Spearman-Brown.

b N/A indicates nonestimable due to floor effect (near-zero variance).

#### Operational Definitions

The key game performance indicators were defined as follows: score was the system-generated composite points; accuracy (%) was the proportion of frames where hotspots were within tolerance; and posture (count) represented the total number of successful hotspot holds. Per movement, the player aligns the left and right controllers and the HMD with 3 predefined hotspots; holding any hotspot for ≥3 seconds is counted as one successful hotspot hold. The 16-movement sequence loops whenever it is completed before the 5-minute limit; therefore, posture is an open-ended count (no fixed cap) within the time window. For interpretability, 48 holds correspond to one full loop (16 movements×3 hotspots).

### Data Preparation and Statistical Analysis

#### Overview

Consistent with the a priori plan, no confirmatory between-condition hypothesis testing (main effects or interaction) was conducted. To enhance transparency, per-condition descriptive summaries (medians and IQRs for the 4 cells) are provided in Table 3.

**Table 3.** Per-condition descriptive statistics.

Outcome^a^	Group A: VR^b^ soothing (n=17), median (IQR)	Group C: VR intense (n=19), median (IQR)	Group B: MR^c^ soothing (n=17), median (IQR)	Group D: MR intense (n=17), median (IQR)
Positive affect	4 (3.8‐4)	4 (3.8‐4)	4 (3.4‐4)	4 (3.8‐4)
Flow	4 (3.2‐4)	3.8 (3.6‐4)	4 (3.2‐4)	4 (3.2‐4)
Competence	3.6 (3.1‐4)	3.8 (3.2‐4)	3.6 (3.4‐4)	4 (3.3‐4)
Challenge	1.4 (1.2‐1.6)	1.4 (1‐1.8)	1.6 (1.1‐1.7)	1.6 (0.8‐1.7)
VRSQ^d^ total	0 (0‐1)	0 (0‐2)	0 (0‐1.5)	0 (0‐2)
Accuracy (%)	75 (56-84)	65 (54-81)	65 (54-77)	79 (54-94)

a Per-condition n shown in column headers. No inferential tests were planned; descriptive summaries only.

b VR: virtual reality.

c MR: mixed reality.

d VRSQ: Virtual Reality Sickness Questionnaire.

#### Data Cleaning

Handled sporadic missingness via median imputation only; no multiple imputation was performed [29].

#### Analysis Plan

Data normality was examined using the Shapiro-Wilk test [30,31]. Most key variables were found not to be normally distributed (Table 4). Nonparametric methods were consequently unanimously selected across hypothesis testing to yield robust and valid inferences. That is, median dimension scores on the GEQ were compared against a theoretical midpoint score of 2.0 using 1-sample Wilcoxon signed-rank tests. Wilcoxon tests correspondingly compared the median against a theoretical score of 0 on the VRSQ symptom scores (rated 0‐3) to determine whether symptoms were distinctly present. With the exploratory nature of this item-level analysis, no adjustment for multiple comparisons was made on these 9 tests. Spearman ρ was used to examine associations between key factors. A level of significance was predetermined at an *α* of .05.

**Table 4.** Shapiro-Wilk test of normality for Game Experience Questionnaire (GEQ) dimensions, Virtual Reality Sickness Questionnaire (VRSQ) symptoms, and game performance indicators.

	W	N	*P* value
Overall GEQ dimension score			
Competence	0.798	70	<.001
Sensory and imaginative immersion	0.777	70	<.001
Flow	0.719	70	<.001
Tension/annoyance	0.275	70	<.001
Challenge	0.974	70	.16
Negative affect	0.573	70	<.001
Positive affect	0.551	70	<.001
VRSQ main symptom			
General discomfort	0.205	70	<.001
Fatigue	0.28	70	<.001
Headache	0.158	70	<.001
Eye fatigue	0.431	70	<.001
Difficulty concentrating	0.153	70	<.001
Head fullness	0.346	70	<.001
Blurred vision	0.349	70	<.001
Dizziness when closing eyes	0.512	70	<.001
Dizziness/vertigo	0.494	70	<.001
Key game performance			
Score	0.967	70	.06
Posture	0.967	70	.06
Accuracy	0.962	70	.03
Average time spent	0.796	70	<.001
Movement distance (m)	0.979	70	.29

#### Overall Usability Analysis

Descriptive statistics summarized the GEQ, the VRSQ, and game log indicators as median (IQR) [19,32].

## Results

### Participant Flow and Analysis Overview

In total, 86 older adults were recruited; 16 (18.6%) discontinued during acclimation due to intolerance, and data from 70 (81.4%) participants were analyzed. Participant flow and group allocation are shown in Figure 1. Per-condition medians (IQRs) for the 4 cells are available in Table 3.

### Scale Reliability

Internal consistency was good for positive affect (*α*=.860; split-half=0.850) and acceptable for the VRSQ total (*α*=.782; split-half=0.689). Competence (*α*=.667; split-half=0.750) and sensory/imaginative immersion (*α*=.657; split-half=0.583) were in the moderate range, whereas flow was borderline (*α*=.546; split-half=0.539). Coefficients for tension/annoyance, challenge, and negative affect were not estimable due to floor effects (near-zero variance; Table 2).

Before the main analysis, the assumption of normality for the key-dependent variables was assessed using the Shapiro-Wilk test. The results are detailed in Table 4. While a few indicators did not significantly deviate from normality (eg, *challenge, P*=.16; game log *score, P*=.06; *posture, P*=.06; *movement distance (m) P*=.29), the majority of variables substantially deviated (eg, *accuracy, P*=.032; most GEQ/VRSQ dimensions, *P*<.001). Therefore, to maintain consistency and ensure robust inference across all analyses, nonparametric procedures were uniformly adopted.

### Descriptive Statistics

We reported per-condition descriptive statistics for key outcomes across the 2×2 design (VR/MR×feedback intensity). As planned, no between-condition inferential tests were conducted; values are summarized as median (IQR) for interpretability with nonnormal distributions (see Table 3 for the 4 cells).

### Overall Usability and Acceptability of the VR Tai Chi Game

To evaluate the overall usability and acceptability of the VR Tai Chi game for older users, this study collected and analyzed survey data from GEQ, VRSQ, and objective game performance log data.

#### Subjective Game Experience (GEQ)

As reflected in Table 5, subjective gaming experience as measured using the GEQ was highly positive. The median levels on positive aspects like “positive affect” (median 4.0), “flow” ( median 4.0), “competence” (median 3.8), and “sensory/imaginative immersion” (median 3.8) all far surpassed the theoretical midpoint of the scale (*P*<.001 in all). Medians on negative aspects such as “tension/annoyance” (median 0) and “negative affect” ( median 0) were below midpoint to a statistically significant degree (*P*<.001 in both). The median level on “challenge” was low to a statistically significant degree as well (median 1.4, *P*<.001).

**Table 5.** Descriptive statistics and one-sample Wilcoxon signed-rank test for Game Experience Questionnaire dimensions.

Dimension	Values, median (IQR^a^)	*P* value^b^
Competence	3.8 (3.2‐4)	<.001
Sensory/imaginative immersion	3.8 (3.2‐4)	<.001
Flow	4 (3.2‐4)	<.001
Tension/annoyance	0 (0‐0)	<.001
Challenge	1.4 (1‐1.6)	<.001
Negative affect	0 (0‐0.25)	<.001
Positive affect	4 (3.8‐4)	<.001

a Game Experience Questionnaire scores were rated on a 0‐4 scale.

b *P* values reflect a one-sample Wilcoxon signed-rank test comparing the median score against the theoretical midpoint of 2.0.

#### Physiological Comfort (VRSQ)

The physiological comfort of the screened participants was assessed using the VRSQ. Overall, the incidence and severity of reported symptoms were low. Detailed item-level medians, statistical test results, incidence rates, and severity scores for reporters are presented in Table 6. The *P* values in the table should be interpreted as exploratory signals of which symptoms were most frequently endorsed, rather than as confirmatory hypothesis tests.

**Table 6.** Overall Virtual Reality Sickness Questionnaire main symptom statistical analysis.

Symptom item	Median	*P* value	Incidence rate (%)	Mean severity for reporters
General discomfort	0 (0-0)	.08	4.3	1
Fatigue	0 (0-0)	.04	7.1	1.4
Headache	0 (0-0)	.16	2.9	1
Eye fatigue	0 (0-0)	.002	15.7	1.273
Difficulty concentrating	0 (0-0)	.18	2.9	1.5
Head fullness	0 (0-0)	.01	10.0	1.286
Blurred vision	0 (0-0)	.01	10.0	1.143
Dizziness when closing eyes	0 (0-0)	<.001	20.0	1.214
Dizziness/vertigo	0 (0-0)	<.001	18.6	1

#### Game Performance

A look at the in-game objective performance data listed in Table 7 reveals wide individual variations among older adult participants. Participants demonstrated a fluid interaction rhythm with a median time spent per action of 6.4 seconds. However, large variations across major indicators were apparent due to wide IQRs seen across score and accuracy measurements (Table 7). Median movement accuracy was 68.5% (IQR 54.2%‐82.3%). Scores higher than 48 indicate that participants completed more than a single loop (or accrued numerous hotspot holds within a movement) within a 5-minute period.

**Table 7.** Key game performance indicators.

Indicator	Values, median (IQR)
Score	96 (70.25-133.5)
Posture^a^ (count; no fixed cap, 48=one loop)	47 (39.75-55)
Accuracy (%)	68.5 (54.2‐82.3)
Average time spent (s)	6.4 (5.5-7.5)
Movement distance (m)	273.5 (231.5-317.25)

a Posture counts the total hotspot holds within the retained 5-min session (best-of-two). The 16-movement sequence loops if completed early.

### Correlation Analysis of Key Usability Factors

Correlation between major usability factors was examined using Spearman rank correlation analysis, and the results were presented in Table 8. Flow was significantly positively correlated with accuracy (ρ=.342; *P*=.004), and competence was significantly positively correlated with accuracy (ρ=.322; *P*=.007). VRSQ total score was significantly inversely correlated to positive affect (ρ=−0.334; *P*=.005). Competence was not significantly correlated with age (ρ=−0.179; *P*=.14).

**Table 8.** Spearman ρ correlation analysis (n=70).

Hypothesis	Variable 1	Variable 2	Correlation coefficient	*P* value
H1	Flow	Accuracy	0.342^a^	.004
H2	VRSQ^b^ total score	Positive affect	−0.334^a^	.005
H3	Accuracy	Competence	0.322^a^	.007
H4	Age	Competence	−0.179	.14

a Correlation is significant at the 0.01 level (2 tailed).

b VRSQ: Virtual Reality Sickness Questionnaire.

## Discussion

### Subjective Experience Profile: High Perceived Competence With Low Perceived Challenge

As presented in Table 5, medians for positive affect, flow, and competence were above the scale midpoint, whereas challenge was markedly below (all *P*<.001). We observe a distinct user experience profile: high perceived competence and low challenge. For older adults who begin using exergames at home, this combination may be most relevant in reducing frustration and promoting first-time engagement [33,34]. At the same time, future research should gradually increase difficulty in an adaptive manner such that motivational support is balanced against accurate skill acquisition in free-living situations [35,36].

The internal consistency profile reveals adequate internal consistency on positive affect and satisfactory consistency on total VRSQ and moderate-to-borderline level in several components of the GEQ. Borderline α on flow is likely owing to constrained variance and the brevity and heterogeneity of item material in this first-stage application involving a single session; such findings on these components should consequently be interpreted correspondingly cautiously.

### Physiological Comfort (VRSQ): Acceptability in Screened Sample and Potential Challenges

Regarding physiological comfort, the VR Tai Chi game demonstrated good tolerability within the screened participant sample, but this conclusion must be interpreted with caution [37]. The initial exclusion of a high 18.6% of recruits due to inability to adapt or strong VR-related sickness signifies a considerable bias in the VR tolerance of the final analyzed sample. Consequently, the currently observed low levels of cybersickness might overestimate the game’s physiological comfort among the general older adult population. Although within the screened “more tolerant” sample, the average reported levels and perceived severity of various physiological discomfort symptoms (mainly mild “dizziness when closing eyes,” “vertigo,” and “eye fatigue”) were low, 15% to 20% of participants still reported these mild symptoms [38]. This implies that cybersickness continues to be a primary barrier that has to be cleared ahead of VR technology deployment to older age categories. This 18.6% exclusion rate translates directly to current technological or design limitations in making VR universally comfortable among older adults and places a realistic limit on such interventions’ immediate impact absent substantive improvements. For plans to promote such VR/MR Tai Chi games to the home environments of community-dwelling older adults, this high initial exclusion rate reveals key practical challenges. In home settings lacking immediate professional guidance, effective user screening, clear adaptation training guidelines, and easy-to-operate personalized comfort settings (eg, mode switching, visual effect adjustments) will be prerequisites for ensuring user safety and continued use [15,39]. To mitigate cybersickness at home, key strategies include starting with MR or low-vection scenes, using brief (≈5 min) sessions, ensuring proper headset fit with stable frame rates, and avoiding artificial locomotion. Furthermore, even mild symptoms, when experienced during unsupervised long-term home use, could affect user experience and adherence, thus necessitating continuous design optimization to minimize discomfort as much as possible [40].

### Accuracy-Competence Paradox

Despite a median movement accuracy of 68.5% (IQR 54.2%‐82.3%), which was below our predefined exploratory benchmark of 80%, participants reported high perceived competence (median 3.8), reflecting a rule set that credits partial hotspot matches (≥1/3 for ≥3 s). This system focuses on older users’ positive affective experiences to improve long-term exercise adherence while reducing frustration and elevating confidence but without concomitant increases in stricter movement norms. However, this dilemma positions an additional discussion regarding such a game’s effectiveness as a very precise device for training within corresponding trade-offs between motivation and skill fidelity.

The permissive rule (≥1 hotspot for ≥3 s) retains motivation but tends to overcredit partial matches. Future iterations will include adaptive multipoint matching and more informative corrective cues to put verifiable and perceived competence into alignment while retaining engagement [41]. It presents a foundational tension: the probable discrepancy between perceived benefit/competence and verifiable physiological/skill-based benefit [42]. While perceived competence is central to adherence, if it is not adequately correlated to correct movement execution, long-term benefit to health can be undermined and signal a need for “verifiable competence” feedback loops that remain motivating. For a mass market product like this, selling independent home-based exercise to older people, such a motivational design can serve to buffer against users experiencing “anxiety alone” if having trouble and will invite continued participation [35]. It does raise a valuable question, though: without professional instruction in a home environment setup, how can it be ensured that movements/movement effectiveness is appropriate? Therefore, future iterations of the gameplay might involve implementing tiered success criteria or optional stepwise movement guidance and feedback modules such that users can reach a personally optimal balance between enjoying playing the game and checking movement correctness in accordance with their own needs [43].

### Insights From Correlation Analysis of Key Usability Factors

The interplay between gameplay performance, user comfort levels, and subjective experience yields direct design recommendations for successful home-based exergames. It was found that “accuracy” was strongly positively correlated with “flow” and “competence.” This indicates that more precise movement performance can guide users into a state of deep, immersive focus, forming a positive feedback loop of “successful execution → flow experience → enhanced participation.” This also offers a more nuanced interpretation of the “accuracy-competence paradox”: the game provides a high baseline of perceived competence for all participants through its lenient judgment, and on top of this, higher accuracy leads to an even stronger sense of competence, cleverly balancing universal motivation with individual rewards. This suggests that users do derive satisfaction from objective mastery; while a safety net of easy success is important, completely removing skill development or clear performance feedback might make the game less engaging long term for some [44,45]. Furthermore, the analysis also highlighted the impact of users’ individual states: the degree of physiological discomfort (VRSQ total score) was significantly negatively correlated with “positive affect.” This warns that even mild discomfort can negatively affect older users’ enjoyment, which is particularly critical in home settings lacking immediate help, thereby re-emphasizing the necessity of minimizing cybersickness risks [46]. Finally, a positive signal was that the study found no significant relationship between “age” and “competence,” suggesting that the game possesses good age inclusivity within the 60 to 75 years age group, and users’ perceived competence did not systematically diminish with increasing age. This has positive implications for its promotion among diverse community-dwelling older adult groups, although caution is advised when generalizing this conclusion to very old adults [39,47].

### Limitations

A number of limitations can be applied to this work. A selection bias in this sample exists since a large number of preliminary participants were lost due to side effects associated with VR, and the resulting group was predominantly female, younger, and physically active. This extremely high level of preexisting physical activity may not be characteristic among this typically sedentary population that such home-based exergames aim to reach but may not serve adequately and hence might limit how much our findings generalize to less active older individuals in this regard. This may inflate physiological comfort but restrict findings’ generalizability to a wider range of heterogeneous older people, especially older individuals who may be less technology-savvy or physically constrained. Finally, a single, brief VR intervention cannot eliminate any novelty effects or long-term adherence or long-term effects on health and retention of skill in a naturalistic home environment.

### Future Direction

Future research will need to proceed beyond virtual-simulated laboratory settings to home-based settings and use strategies such as Ecological Momentary Assessment to assess long-term use, adherence, skill learning, and actual resultant health effects. It will be important to diversify the samples of participants to include a broader age range, a wider range of health statuses, and a wider range of technology backgrounds to assess the generalizability of such findings. Studies will need to work toward developing adaptive VR systems capable of shifting difficulty and feedback in a dynamic fashion, dependent upon user progress. We will assess fixed versus adaptive thresholds and include per-user calibration routines within a home-based study.

This will aid in connecting, keeping participants motivated, and ensuring appropriate skill acquisition to promote successful unsupervised home-based practice. Although this current system used the Meta Quest 3 headset and hand tracking, future work could include alternative systems (eg, Xbox/Kinect) or outward-facing full-body tracking systems to better assess posture in unsupervised home-based settings. Ecological Momentary Assessment is described as a future longitudinal assessment methodology to assess adherence and in-the-wild engagement but not a single-session assessment of emotion.

### Conclusions

This study demonstrates that well-designed VR/MR Tai Chi exercises can be a realistic and inspiring alternative exercise among community-dwelling older adults, supporting aging in place initiatives. A self-reported profile of high competence but low challenge perception implies early initiation but will need variable difficulty to maintain motivation in alignment with acuity of therapy for long-term use at home. Although physiologically tolerable in this prescreened population, a high entry exclusion rate due to cybersickness is a major barrier to wider use. A future direction to wider use success is a balance between motivational design of content and therapy acuity and modifications to system usability and comfort to provide net health gains in homeowner unsupervised use.

## Abbreviations

GEQ: Game Experience Questionnaire
HMD: head-mounted display
MDA: mechanics, dynamics, and aesthetics
MR: mixed reality
VR: virtual reality
VRSQ: Virtual Reality Sickness Questionnaire
